# Liquid‐Phase Assisted Engineering of Highly Strong SiC Composite Reinforced by Multiwalled Carbon Nanotubes

**DOI:** 10.1002/advs.202002225

**Published:** 2020-09-21

**Authors:** Yuchi Fan, Erhong Song, Tufail Mustafa, Ruicong Liu, Pengpeng Qiu, Weiwei Zhou, Zhenxing Zhou, Akira Kawasaki, Keiichi Shirasu, Toshiyuki Hashida, Jianjun Liu, Lianjun Wang, Wan Jiang, Wei Luo

**Affiliations:** ^1^ State Key Laboratory for Modification of Chemical Fibers and Polymer Materials College of Materials Science and Engineering Donghua University Shanghai 201620 China; ^2^ Institute of Functional Materials Donghua University Shanghai 201620 China; ^3^ State Key Laboratory of High Performance Ceramics and Superfine Microstructure Shanghai Institute of Ceramics Chinese Academy of Sciences Shanghai 200050 China; ^4^ Department of Chemical Engineering Balochistan University of Information Technology Engineering and Management Sciences (BUITEMS) Quetta 87300 Pakistan; ^5^ Department of Materials Processing Graduate School of Engineering Tohoku University Sendai 980‐8579 Japan; ^6^ Department of Aerospace Engineering Tohoku University Sendai 980‐8579 Japan; ^7^ Fracture and Reliability Research Institute Tohoku University Sendai 980‐8579 Japan

**Keywords:** ceramic composites, liquid‐phase sintering, multiwalled carbon nanotubes, strengthening

## Abstract

Despite the ultrahigh intrinsic strength of multiwalled carbon nanotube (MWCNT), the strengthening effect on ceramic matrix composite remains far from expectation mainly due to the weak load transfer between the reinforcement and ceramic matrix. With the assistance of the in situ pullout test, it is revealed that the liquid‐phase sintering (LPS) can serve as a novel strategy to achieve effective load transfer in MWCNT reinforced ceramic matrix composites. The YAlO_3_ formed liquid phase during spark plasma sintering of SiC composite greatly facilitates radical elastic deformation of MWCNT, leading to highly increased interfacial shear strength (IFSS) as well as interlayer shear resistance (ISR) of nested walls. The liquid phase with superior wettability can even penetrate into the defects of MWCNT, which further increases the ISR of MWCNT. Moreover, the first‐principles calculation indicates that the oxygen terminated YAlO_3_ phase displays much stronger bonding compared with SiC matrix, which is also responsible for the large IFSS in the composite. As a result, as high as 30% improvement of bending strength is achieved in the composite with only 3 wt% MWCNT in comparison to the monolithic ceramic, manifesting the unprecedented strengthening effect of MWCNT assisted by LPS.

## Graphical abstract

The liquid‐phase sintering can significantly improve the load transfer between multiwalled carbon nanotube and SiC ceramic matrix by increasing the interfacial shear strength and interlayer shear resistance, giving rise to unprecedented strengthening effect of 30% with filler fraction of only 3 wt%. The finding provides a strategy for designing highly strong ceramics reinforced by low‐dimensional reinforcements.

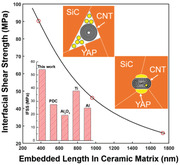

With exceptional stiffness, strength, and chemical stability, carbon nanotube (CNT) has been widely considered as one of the most promising reinforcements for engineering materials since its discovery.^[^ ^1^, ^2^, ^3^ ^]^ However, the strengthening effect of CNT, either with single‐walled (SWCNT) or multiwalled (MWCNT) structure, is usually found to be rather limited in composites, considering the ultrahigh intrinsic strength of CNT.^[^ ^4^, ^5^ ^]^ In the case of SWCNTs, the mechanical properties of composite is mainly restrained by the weak modulus and strength of the bundles of SWCNT rather than individual tubes,^[^ ^6^ ^]^ because breaking bundles into individually dispersed tubes in the matrix is extremely difficult based on the current technology. In this sense, MWCNTs are much more preferable in terms of dispersion due to the relatively lower surface area and larger radial diameter. The load transfer of MWCNTs in composite can thus be effectively improved through surface modification especially for the polymer matrix composites (PMC), mainly benefiting from the high interfacial shear strength (IFSS).^[^ ^7^ ^]^ In addition, highly straight and aligned MWCNTs with large volume fraction can also be achieved in PMC via stretching technology, which gives rise to the significantly enhanced strength.^[^ ^8^ ^]^

However, most of the processing strategies that work for polymers are difficult to be applied for ceramic matrix composites (CMC) because of the poor wetting behavior between rigid ceramic powder and CNT. Although the dispersion of MWCNT in CMC has been greatly improved by the advanced technologies developed in recent years,^[^ ^9^, ^10^, ^11^, ^12^, ^13^ ^]^ realizing high IFSS in engineering ceramics remains very challenging due to the lack of chemical bonding between CNTs and matrix.^[^ ^14^ ^]^ More importantly, the low interlayer shear resistance (ISR) which stems from the nested tubes bonded by weak van der Waals force can result in fracture of outmost walls yet intact of most inner walls under loading, leaving the so‐called “sword‐in‐sheath” (SIS) structure with inferior strengthening effect.^[^ ^15^, ^16^ ^]^

Therefore, to obtain high strength in MWCNT reinforced CMC, the microstructural design should be conceived from three aspects. First, the IFSS between MWCNT and ceramic matrix should be enhanced to prevent the facile pullout of tube. Although pullout is beneficial to improve the toughness of composite, facile pullout without effective load transfer only leads to both low strength and toughness in the composite. Second, the ISR should be increased to fully exploit the load bearing capacity of individual MWCNT. Third, processing method to force the alignment of MWCNTs in composite along the direction of tensile stress should be explored. Herein, taking *α*‐SiC as a typical example of engineering ceramic, we found that the abovementioned targets can be achieved simultaneously with the assistance of liquid‐phase sintering (LPS).

The mechanical properties of MWCNT‐reinforced CMC greatly rely on the dispersion of second phase. In this study, the homogeneous mixing of composite powder was achieved by a self‐assembly protocol, in which surface modification for both MWCNTs and ceramic powder was conducted. As demonstrated by previous research,^[^ ^17^ ^]^ the acid treatment of MWCNT is very effective for grafting hydrophilic groups to the sp^2^ carbon bonds, which highly improves the dispersibility of MWCNTs in water. However, it has to be noticed that the acid treatment has some side‐effect that could undermine the structure of MWCNTs inevitably. It is observed that while the length of MWCNTs was not greatly shortened (**Figure** **1**a), the notched outmost walls can be readily found after acid treatment (Figure 1b), indicating the etching effect from corrosive acid. Although some researches have demonstrated the benign effect of these notches on improving load transfer, special attention was paid to balance the wettability of MWCNTs in water and the damage to walls which could be detrimental to the intrinsic strength. After acid treatment, it can be seen that the MWCNTs exhibited a negative surface charge (Figure 1c) while preserved the highly crystalline hollow structure almost as same as that of the pristine material (Figure 1b). On the other hand, to deliver the liquid‐sintering of SiC ceramic at low temperature, Y_2_O_3_ and Al_2_O_3_ were added as the sintering additives for their low eutectic point,^[^ ^18^, ^19^ ^]^ which could be readily mixed with commercial *α*‐SiC powder via ball milling. However, the surface charges of *α*‐SiC and Y_2_O_3_ powder are both negative that could not trigger the self‐assembly process (Figure S1, Supporting Information). Thereby, the mixed ceramic powder was modified by silane coupling agent to render positive surface charge (Figure 1c), noting that the similar large zeta potential obtained for each type of ceramic powder is critical to prevent the premature precipitation when mixing with MWCNTs. Accordingly, possessing the opposite surface charge at low pH (≈2), dropwise addition of MWCNT dispersion into the colloid of ceramic powder led to the simultaneous assembly of particles to form a homogeneous aggregation of hybrid powder (Figure 1d).

**Figure 1:**
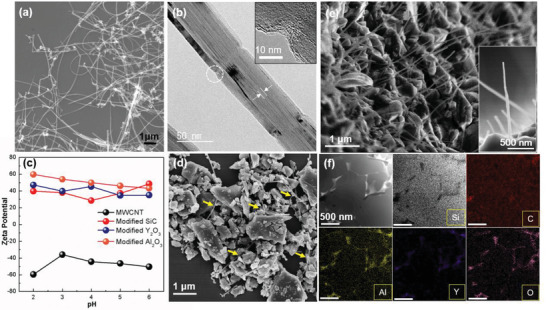
Preparation of MWCNT/SiC Composite. a) SEM image of the acid‐treated MWCNTs. b) TEM image of a single acid‐treated MWCNT with a hollow core, the inset indicates the notch on outmost walls induced by acid treatment. c) Zeta potential of MWCNT and ceramic powders after modification. d) SEM image of MWCNT/SiC hybrid powder with homogeneous dispersion of MWCNTs. e) The SEM image of fracture surface for bulk composite; the inset shows an individual protruding MWCNT with the length of ≈2 µm. f) Dark field STEM image of the composite and corresponding TEM‐EDS mapping in the same area for Si, C, Al, Y, and O, respectively.

The well‐mixed hybrid powder was consolidated by spark plasma sintering (SPS) to obtain fully dense bulk composite. The LPS with high‐pressure and fast heating rate during SPS can accomplish the densification to be completed at relatively low temperature (1800 °C) in short time, which is beneficial to protect the MWCNTs from damage. After sintering, the protruding MWCNTs with exposed length of ≈2 µm can be readily recognized on the fracture surface of composite under SEM observation (Figure 1e), indicating the highly preserved structure. It is also observed that the MWCNTs are uniformly distributed in ceramic matrix without apparent clusters, which is even comparable to the dispersion of some PMCs.^[^ ^20^, ^21^ ^]^ Meanwhile, the ceramic matrix displays a typical microstructure of LPS SiC, where the equiaxed SiC grains are closely compacted with intergranular phase mainly concentrated on the triple junctions. The TEM‐EDS mapping reveals the concentration of Al, Y, and O in the intergranular phase (Figure 1f), suggesting the reaction of Al_2_O_3_ and Y_2_O_3_ to form the liquid phase during the sintering process.

The composition of liquid phase in matrix was further identified by XRD analysis (**Figure** **2**a). Despite of the weak intensity compared with predominant *α*‐SiC (6H‐SiC) phase (PDF:49‐1428), the peaks in consistent with perovskite‐like YAlO_3_ (YAP, PDF: 33‐0041) still can be distinguished. Meanwhile, trivial garnet phase (Y_3_Al_5_O_12_,YAG) may also exist since it is more thermodynamically stable.^[^ ^22^ ^]^ From the XRD patterns, it is difficult to locate the peaks belonging to MWCNTs because of the lower fraction (3 wt%) with respect to the YAP phase (≈5 wt%). Therefore, we exploited Raman spectroscopy as a powerful tool to investigate the influence of processing and sintering on MWCNTs (Figure 2b). The quality of MWCNT was first evaluated from the intensity ratio of D band at ≈1350 cm^−1^ and G band at ≈1580 cm^−1^ (*I*_D_/*I*_G_). The pristine MWCNT shows a small value of *I*_D_/*I*_G_ around 0.33, which increases slightly to 0.42 for the acid‐treated MWCNTs due to the defects induced by the etching effect. Interestingly, the *I*_D_/*I*_G_ decreases to 0.37 inversely for the MWCNT in composite, which indicates the recovery of sp^2^ bonds at the sintering temperature. Note that the sintering process can be considered as same as high‐temperature annealing at 1800 °C in vacuum.^[^ ^23^ ^]^ More importantly, the deformation of MWCNTs in the ceramic environment can be monitored by the 2D band in Raman spectra as well.^[^ ^24^ ^]^ The 2D band in sp^2^ carbon materials represents a second‐order two‐phonon process which is very sensitive to the phonon structure perturbation.^[^ ^25^ ^]^ The shift of 2D band to higher or lower wavenumbers clarify that whether MWCNTs are in tensile or compressive strain, respectively.^[^ ^26^, ^27^ ^]^ In MWCNT/SiC composite with 3 wt% filler loading, the 2D band upshifts from 2696 to 2703 cm^−1^ compared to pristine MWCNTs, which indicates that MWCNTs are under great compressive strain in the composite. This phenomenon, which was ascribed to the thermal expansion induced residual stress in MWCNT reinforced CMC before,^[^ ^9^ ^]^ will be discussed in details later.

**Figure 2:**
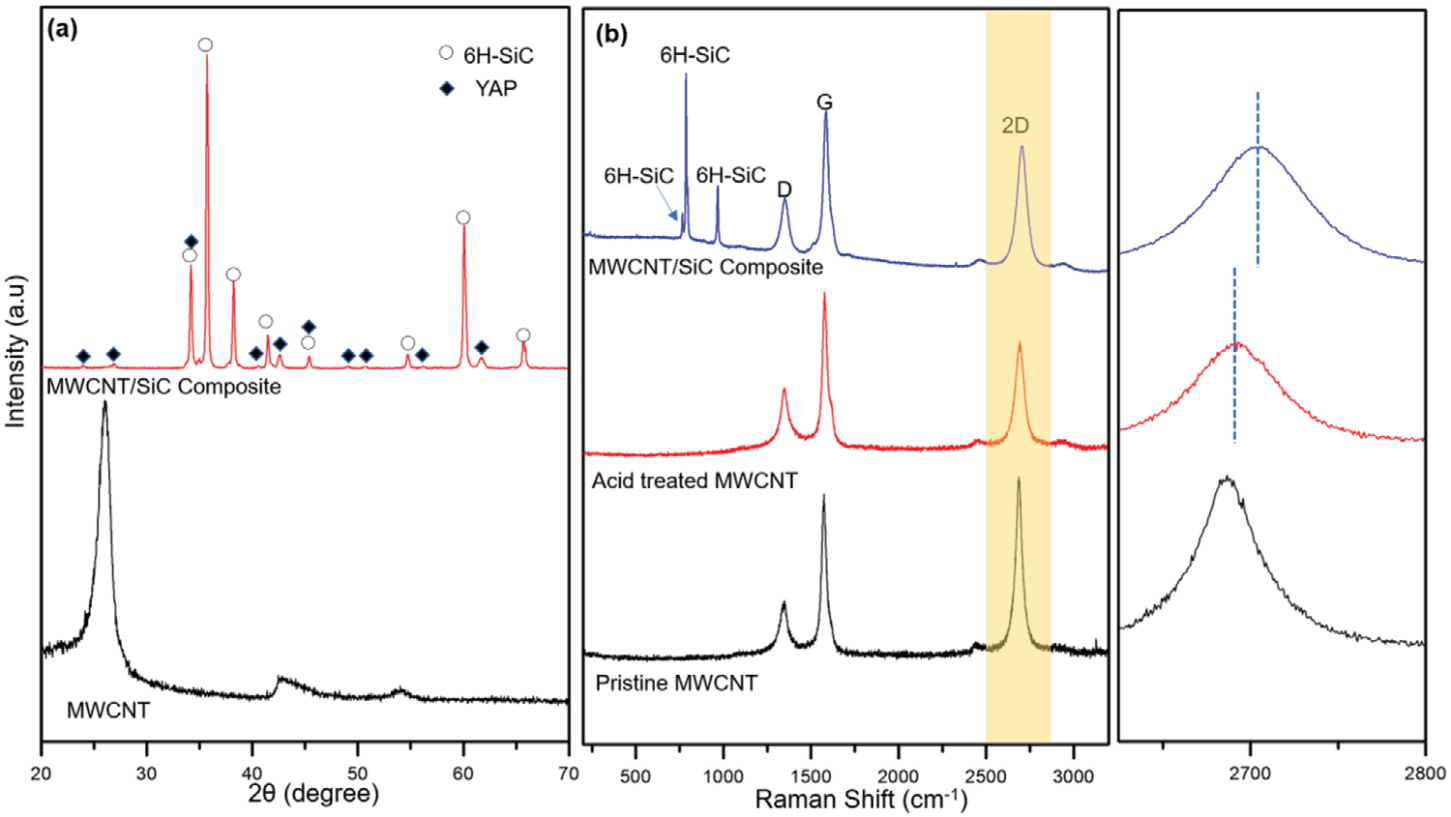
a) XRD patterns of MWCNT and the MWCNT/SiC composite with 3 wt% filler content, the *α*‐SiC and liquid phase can be assigned to 6H‐SiC(PDF: 49‐1428) and YAG(PDF: 33)YAP(PFD: 33‐0041), respectively. b) Raman spectra for the pristine MWNCT, acid‐treated MWCNT, and composite, respectively; the enlarged figure shows the shift of 2D bands.

With the homogenous dispersion of MWCNTs in SiC matrix, we investigated the mechanical properties of MWCNT/SiC composite and monolithic SiC. The strength was evaluated by the modified small punch (MSP) test (Figure S2, Supporting Information), which is a biaxial bending technique giving flexural strength value comparable to the traditional 4‐point bending, but with higher reliability owing to the insensitivity to edge machining.^[^ ^28^ ^]^ In this study, the MSP strength for monolithic SiC is similar to the reported value (>450 MPa) for the LPS *α*‐SiC tested by 4‐point bending,^[^ ^29^ ^]^ further validating the results obtained here. Strikingly, the SiC composite with only 3 wt% MWCNT shows an MPS strength of ≈659 MPa, which is 31% higher than that of the monolithic SiC (**Table** **1**). Noting that the improvement achieved by MWCNT in CMC is normally 10–20% so far,^[^ ^30^, ^31^ ^]^ the high strength realized here implies unprecedented strengthening effect from MWCNT in the SiC composite. Here we argue that the possibly restrained grain growth has very limited strengthening effect compared to MWCNT, which can be proved by the much lower MSP strength of composite with removed MWCNT (Figure S3, Supporting Information). By contrast to the enhanced strength, the Young's modulus experienced dramatic decrease upon addition of 3 wt% of MWCNT, which leads to the increase of nominal strain‐to‐failure to as large as 0.22%. Although the increased strain‐to‐failure has been found before in CMCs reinforced by low‐dimensional carbon material such as MWCNT and graphene,^[^ ^9^, ^32^ ^]^ the simultaneous achievement of such high strength and strain‐to‐failure is unusual. Given that the large strain‐to‐failure has been found in SiC before when highly aligned cellular structure exists,^[^ ^33^ ^]^ this phenomenon should be ascribed to the highly anisotropic alignment of MWCNT in SiC matrix, which is critical to the strengthening effect as well. Indeed, the difference between in‐plane and out‐of‐plane electrical conductivity is very large in the composite (Table 1), strongly indicating the oriented MWCNT along in‐plane direction.

**Table 1:** Basic information and mechanical properties of MWCNT/SiC composite

Filler content [wt%]	Density [g cm^−3^]	Relative density [%]	MSP strength [MPa]	Young's modulus [GPa]	Nominal strain‐to‐failure [%]	Vickers hardness [GPa]	Indentation toughness [MPa m^1/2^]	Electrical conductivity [Sm^−1^]
0	3.24	98.53	504.8 ± 9.5	430.9	0.12	18.4 ± 0.4	4.0 ± 0.6	–
3	3.17	98.39	659 ± 16.4	298.6	0.22	16.8 ± 1.6	4.9 ± 0.6	390^a)^
								4.9^b)^

a) In‐plane DC electrical conductivity;

b) Out‐of‐plane AC electrical conductivity at 15 kHz.

To unveil the mechanism behind the highly improved strength in the composite, we investigated the IFSS in composite by the in situ pullout test. The protruding MWCNT on the facture surface was first selected and clamped on the tip of cantilever via a motor stage equipped in SEM, and pulled until leaving the matrix or failure (see the Supporting Information for details). The loading force obtained from the deformation of cantilever can be used to calculate ether the average IFSS (*τ*_avg_) in the case of pullout, or nominal tensile strength of MWCNT (*σ*_N_) when failure occurs, using the following equation

(1) $$\tau_{\mathrm{avg}}=\frac{F_{\mathrm{pull}}}{\pi DL_{\mathrm{emb}}}$$

(2) $$\sigma_{N}=\frac{F_{\mathrm{pull}}}{\pi {\left(\frac{D}{2}\right)}^{2}}$$

where *D* is the diameter of MWCNT and *L*_emb_ is the embedded length of MWCNT. Since gold coating was made before in situ pullout test, the protruding part and embedded part of MWCNT can be precisely determined from TEM image. Remarkably, through three qualified pullouts of MWCNT out of total nine tests (Figure S4, Supporting Information), an average IFSS of 54.1 ± 32.2 MPa can be obtained for the composite, which is significantly higher than the value in Al_2_O_3_ matrix (19.2 ± 6.6 MPa),^[^ ^34^ ^]^ polymer derived ceramic matrix (27.5 ± 5.8 MPa),^[^ ^35^ ^]^ as well as many metal matrix composites (**Figure** **3**a). Moreover, the average IFSS increases with decreasing embedded length of MWCNT, which is completely consistent with the shear‐lag theory.^[^ ^36^ ^]^ Accordingly, the measured IFSS can be fitted by the following equation

(3) $$\tau_{\mathrm{avg}}=\tau_{\max}\frac{\tanh\left(\beta L_{\mathrm{emb}}\right)}{\beta L_{\mathrm{emb}}}$$

where *τ*_max_ is the maximum IFSS on the nanotube and *β* is a shear‐lag constant. The fitted value for *τ*_max_ is 117.26 MPa, reaching the value for surface modified MWCNT/epoxy composite (*τ*_max_ =151 MPa) in which the interface is strongly bonded by ester groups. The large IFSS can not only improve the load transfer between MWCNT and ceramic matrix, but also help to dissipate the energy during crack propagation, as evidenced by the improved indentation toughness of ≈22% in this composite (Table 1).

**Figure 3:**
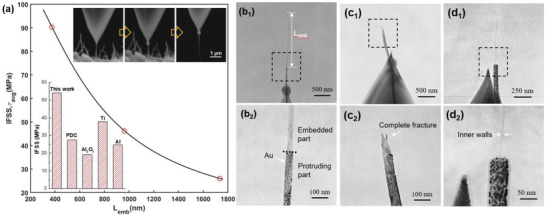
a) The correlation between IFSS and embedded length (*L*_emb_) of MWCNT determined via in situ pullout test, the black curve is the fitting result based on Equation (3). The right top set illustrates the approaching, clamping, and pullout during an in situ test, the left down inset indicates the comparison of IFSS for various matrixes. b_1_) TEM images of a typical pullout MWCNT, showing the distinguishable embedded and protruding part in (b_2_). c_1_,c_2_) TEM images of typical fractured MWCNT with complete broken walls. d_1_) TEM images of fractured MWCNT of SIS type, showing the very thin remaining inner walls after fracture in (d_2_).

Apart from the pullout nanotubes, the other MWCNTs underwent ether complete or SIS type fracture (Figure 3b,c). The calculated *σ*_N_ scattering from 0.58 to 3.4 GPa is almost identical to the measured fracture strength for MWCNT without acid treatment (≈2 GPa),^[^ ^37^ ^]^ reflecting very limited damage induced after acid treatment and sintering. Moreover, unlike the SIS fracture observed in the other MWCNT reinforced CMC,^[^ ^15^, ^38^ ^]^ the unbroken inner walls (the “sword” part) in the SiC composite here typically exhibit only a few layers. As shown in Figure 3d, the diameter of MWCNT and unbroken inner walls is respectively 8.12 and 54.8 nm, which means most of walls in the tube failed during the in situ pullout test. Therefore, it can be inferred that the ISR of MWCNTs in the composite must be sufficiently high to guarantee the load transfer from outer to inner walls and thus inhibit the premature failure of MWCNTs. In addition, if we calculate the nominal tensile strength for the pullout MWCNTs as well (Table S1, Supporting Information), it is found that the *σ*_N_ (≈3.68 GPa) is apparently higher than that of fractured MWCNTs, which reveals the fact that the pullout took place only when the intrinsic tensile strength for the individual MWCNT is high, otherwise multiwalled fracture would occur as a consequence of highly effective load transfer in the composite.

On the basis of the above results, it can be seen that both high IFSS and ISR have been realized in the MWCNT/SiC composite, which is one of the most important reasons for the remarkable strengthening effect. Considering the large compressive strain in MWCNT detected by Raman spectroscopy, it is very obvious that the high IFSS and ISR are closely related to the residual stress, which is usually ascribed to the difference of coefficient of thermal expansion (CTE) between MWCNT and ceramic matrix.^[^ ^39^, ^40^ ^]^ However, the MWCNT actually possesses a positive radial CTE in the range of 1.6 × 10^−5^–2.6 × 10^−5^ K^−1^,^[^ ^41^, ^42^ ^]^ which is roughly one‐order larger than that of ceramic materials including *α*‐SiC,^[^ ^43^ ^]^ Al_2_O_3_,^[^ ^44^ ^]^ and YAP.^[^ ^45^ ^]^ Accordingly, there must be other reason that leads to the large redidual stress.

To clarify the reason for the residual stress, we further investigated the microstructure of MWCNT/SiC composite by TEM and STEM. As shown in **Figure** **4**a_1_, two MWCNTs aligned along the triple junction are surrounded by the liquid phase with very clear contrast to the ceramic matrix. The gathering of MWCNTs at triple junction here is a natural consequence of LPS rather than agglomeration for the composite with 3 wt% filler content. Selective area electron diffraction (SAED) confirmed the liquid phase of YAP and matrix phase of 6H‐SiC. It can be seen that the wetting between YAP phase and MWCNT is very good that allows the liquid phase to fill the whole space between MWCNTs and SiC. On the other hand, the nanotubes shows highly wrinkled interlayer structure without hollow cores on the cross‐section (Figure 4c_1_,c_2_) in contrast to the perfectly paralleled structure in freestanding MWCNT, strongly indicating the high compressive stress exerted to the graphitic walls. Note that while MWCNT has extremely large elastic modulus along axial direction, the elastic modulus in radial direction could be quite small (0.3–4 GPa) while the reversible deformability is large (up to 40%).^[^ ^46^ ^]^ Therefore, the radial elastic deformation could occur easily by the applied pressure during SPS. However, the compressive stress transfer turns out to be inefficient at the triple junctions, where MWCNTs are yet prone to gather during consolidation process. As shown in Figure 4c, only mild deformation can be seen in MWCNTs at the triple junctions without liquid phase. In addition, the compressive stress seems only from the directions of contacted grains, leading to delamination and kink of walls. By contrast, when the liquid phase exists at triple junctions, the large pressure from the surrounding SiC grains can transfer to the nanotubes effectively, which gives rise to the highly wrinkled layers and disappeared hollow core. The induced radial elastic deformation in MWCNT can be preserved with rapid solidification of liquid YAP, because of the much higher elastic modulus in solid YAP (318 GPa, RT) and *α*‐SiC (373.6 GPa at 1800 K).^[^ ^43^, ^47^ ^]^ The different structure in MWCNT induced by LPS can be illustrated by Figure 4d_1_,d_2_.

**Figure 4:**
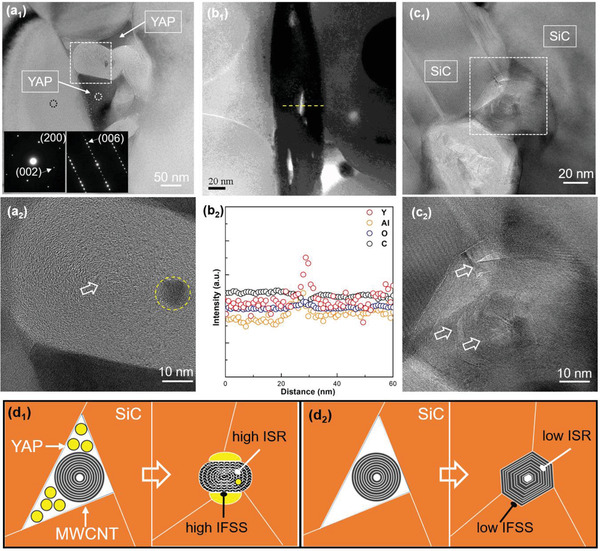
a_1_) TEM image of the triple junction containing liquid phase and MWCNTs in the composite; the insets are SAED patterns of YAP phase (white dot) and SiC matrix (black dot), respectively. a_2_) The HRTEM image for the square area in (a_1_), showing the highly wrinkled layers and disappeared hollow core; the white arrow denotes the lack of hollow core in the center, and the yellow circle denotes a nanoparticle inside the compressed walls. b_1_) Dark field STEM image illustrating the needle‐like nanoparticles inside MWCNT. b_2_) EELS analysis along the yellow line indicated in (c_1_), showing the elemental concentration of Y, Al, and O with respect to C. c_1_) TEM image of the triple junction containing MWCNTs but without liquid phase (c_2_). The HRTEM image shows the kink structure and hollow core (white arrows) in the square area of (c_1_). d_1_) Schematic illustration of the sintering process of an MWCNT included junction with YAP phase (yellow particles), and d_2_) the case without liquid phase.

Furthermore, it is very surprising to find that some embedded particles exist inside the walls of tightly compressed MWCNT (Figure 4a_2_). STEM observation from the lateral side of MWCNT illustrates that these nanoparticles are actually needle‐like along the axial direction of nanotube (Figure 4b_1_). The electron energy loss spectra (EELS) analysis clearly shows the elemental concentration of Y, Al, and O in the needle‐like particles (Figure 4b_2_), leading to the conclusion that it is actually the liquid phase that was forced to penetrate into the defects and channels of MWCNT by the high pressure during LPS. This phenomenon, which has not been observed in any MWCNT included composite before, reflects again the good wetting of liquid phase and high compressive stress applied onto the MWCNT. During the cooling process, the liquid YAP solidified quickly to form the rigid needle‐like particles that can effectively pin the movement between layers under loading, further improving the ISR of MWCNT. Thus, the LPS can not only greatly enhance the IFSS by increased friction force, but also facilitate the increase of ISR through the highly wrinkled structure and embedded nanoparticle pining.

Finally, although there is no noticeable reaction between MWCNT and YAP phase by TEM observation, it is still possible that interfacial bonding can be generated under the environment of high pressure and temperature, which could influence the IFSS in the composite. Therefore, we carried out DFT calculations to further investigate the interfacial bonding formation between MWCNT and YAP phase. For comparison, the relaxed structure of multiple interfaces including MWCNT/YAP‐O (O terminated (002)), MWCNT/YAP‐Y (Y terminated (002)), MWCNT/SiC‐Si (Si terminated (0001)), and MWCNT/SiC‐C (C terminated (0001)) have been constructed (**Figure** **5**a–d; Figure S5, Supporting Information), where the inherent wrinkle and distorted outmost wall of MWCNT is due to the interaction between MWCNTs and YAP/SiC surfaces. The corresponding calculated charge density differences of four different interface structures are also represented (Figure 5e–h), qualitatively reflecting the redistribution of electron states of atoms at the interfaces. It can be seen that the amount of electron states of MWCNT/YAP(002) interface is much more than that of MWCNT/SiC (0001) interface, owing to the difference in electronegativity of the interface. As indicated by the charge redistribution of C atoms at MWCNT/YAP (002) interface, the amount of electronic states of C atoms at MWCNT/YAP‐O interface is relatively increased compared with MWCNT/YAP‐Y interface due to the larger electronegativity of O‐terminated interface. In addition, the average interaction energy (Δ*E*) values for four types of interfaces are calculated to range from −0.063 to −0.287 eV per atom (Figure 5i). Particularly, the MWCNT surface exhibits a stronger bonding strength of ≈−0.287 eV per atom when connected with the active O terminated YAP surface, and the Δ*E* of MWCNT/YAP is apparently larger than that of MWCNT/SiC, which firmly supports the stronger interface in the presence of YAP phase. In addition, the formation energy of carbon vacancy around the MWCNT/YAP‐O interface was calculated as shown in Figure S6 of the Supporting Information. For the interface model of MWCNT/YAP, the surface energy of YAP (002)‐O/Y was also calculated to further research the stability of surface. As shown in Figure S7 of the Supporting Information, it is found that the surface of YAP (002)‐O exhibits not only higher surface activity but also relatively stronger stability due to the lowest value of surface energy.

**Figure 5:**
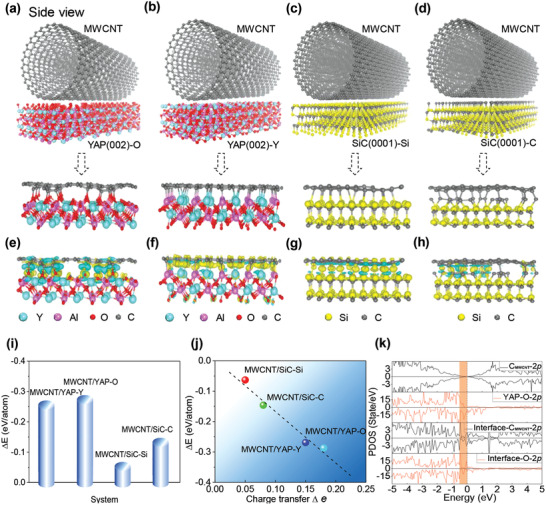
Calculated interaction energy and electronic structures of multiple interfaces between MWCNT and YAP/SiC surface. a–d) The optimized structure of interfaces: MWCNT/YAP(002)‐O (O terminated), MWCNT/YAP(002)‐Y (Y terminated), MWCNT/SiC (0001)‐Si (Si terminated), MWCNT/SiC(0001)‐C (C terminated). e–h) The corresponding charge density differences of different interfaces; the yellow and blue regions indicate the accumulated or dispersed amount of electron states of atoms around the interface, respectively. i)The average interaction energy Δ*E* of multiple interfaces between MWCNT and YAP/SiC surface. j) Linear correlation between Δ*E* and the average amount of charge transfer Δ*e* of MWCNT in different YAP/SiC surface. k) Projected density of state (PDOS) of C_MWCNT_‐2p orbital, YAP(002)‐O‐2p orbital, C‐2p orbital of MWCNT/YAP(002)‐O, O‐2p orbital of MWCNT/YAP(002)‐O.

The analysis of Bader charge of carbon atoms of MWCNT (C_MWCNT_) at different interfaces shows that there exists a linear correlation between the Δ*E* and the amount of charge transfer of C_MWCNT_ atoms, suggesting that the strong bonding between MWCNT and YAP phase is mainly attributed to the large amount of electrons transfer at the interface, while the small amount of electrons transfer can hardly lead to the formation and strengthening of interface. As shown in Figure 5k and Figure S8 (Supporting Information), the projected density of states (PDOS) for multiply interface is revealed to further understand the strengthening effect at interface. For the MWCNT/YAP (002)‐O interface, the amount of electronic states near Fermi level (−0.5 to 0 eV highlighted by orange rectangular areas) determines the interaction energy of interface. At the −0.5 to 0 eV energy level, the electron states of C_MWCNT_‐2p orbital in free MWCNT around Fermi level preferentially hybridize with O‐2p orbitals in free YAP‐O in order to form the interface, inducing more extra free electrons transfer and thus enhanced strength of C—O bond. The electron states of O‐2p orbitals in free YAP‐O between −0.5 and 0 eV are dragged to the deep energy level (−3 to −2 eV) due to hybridizing with the C_MWCNT_‐2p orbital in free MWCNT. After interface formation, the electron states of C_MWCNT_‐2p increase while that of O‐2p orbital decrease at MWCNT/YAP‐O interface apparently around Fermi level, which facilitates the charge redistribution. Meanwhile, the formation of C—O bond can also promote thermodynamic stability of interface.

In summary, we fabricated the highly strong MWCNT/SiC composite via self‐assembly strategy with the assistance of LPS. The liquid phase facilitated the alignment of MWCNT during hot pressing, which is beneficial to the improvement of strength and strain‐to‐failure in composite. The in situ pullout test and analysis of failure modes for MWCNTs indicate that large IFSS (≈54 MPa) and ISR have been achieved in the composite, which accounts for the effective load transfer at interface. The investigation of microstructure proves that the existence of liquid phase under hot pressing effectively increased the transfer of compressive stress to the MWCNT, leading to the large residual stress derived from radial elastic deformation. The highly wrinkled layer and infiltrated liquid phase can also greatly improve the ISR. Moreover, the first‐principles calculation indicates that the oxygen terminated YAlO_3_ phase displays much stronger bonding compared with SiC matrix, which is also responsible for the large IFSS in the composite. Noting that most structural ceramic materials can be obtained by LPS in realistic application, our findings proposed a completely novel strategy for designing the high‐strength CMCs reinforced by MWCNT as well as other low‐dimensional materials.

## Conflict of Interest

The authors declare no conflict of interest.

## Supporting information

Supporting Information
