# Supplementary material for: Liquid‐Phase Assisted Engineering of Highly Strong SiC Composite Reinforced by Multiwalled Carbon Nanotubes
Source: Adv Sci (Weinh). 2020 Sep 21;7(21):2002225. doi: 10.1002/advs.202002225 (PMC7610309; doi:10.1002/advs.202002225)
Supplement: Supplementary file 1 — Supporting Information [file ADVS-7-2002225-s001.pdf]

© 2020 Wiley-VCH GmbH

ADVANCED SCIENCE
Open Access

Supporting Information

for *Adv. Sci.,* DOI: 10.1002/advs.202002225

Liquid-phase assisted engineering of highly strong SiC composite reinforced by multi-walled carbon nanotubes

Yuchi Fan, Erhong Song, Tufail Mustafa, Ruicong Liu, Pengpeng Qiu, Weiwei Zhou, Zhenxing Zhou, Akira Kawasaki, Keiichi Shirasu, Toshiyuki Hashida, Jianjun Liu*, Lianjun Wang, Wan Jiang and Wei Luo**

© 2020 Wiley-VCH GmbH

Supporting Information

Liquid-phase assisted engineering of highly strong SiC composite reinforced by multi-walled carbon nanotubes

Yuchi Fan, Erhong Song, Tufail Mustafa, Ruicong Liu, Pengpeng Qiu, Weiwei Zhou, Zhenxing Zhou, Akira Kawasaki, Keiichi Shirasu, Toshiyuki Hashida, Jianjun Liu*, Lianjun Wang, Wan Jiang and Wei Luo**

1. **Experimental procedure**

1.1 Acid treatment of MWCNTs

0.3g MWCNTs were placed in a three-neck flask and mixed with a 100 ml concentrated acid solution of 98% sulfuric acid (H_2SO_4) and 68% nitric acid (HNO_3) with volume ratio (3:1), respectively. Mixture was placed in ultrasonic bath for 30 min, then refluxed at 110 ^oC for 20 min with vigorous stirring. After cooling it to room temperature, 200 ml deionized water was added to dilute the mixture. The obtained MWCNTs were repeatedly washed by vacuum filtration with a large amount of deionized water until pH became neutral. Finally, the MWCNTs cake was dried for 12 h at 80 ^oC.

1.2 Surface modification of SiC powder and sintering aids

SiC powder (Saint-Gobain Co) was first mixed with 5wt.% Y_2O_3 (H.C STARK Co.) and 2wt.% Al_2O_3 (Sumitomo chemicals) as sintering aid by using planetary ball mill. The surface modification of mixed powder (1g) was then conducted by refluxing in toluene (100 ml) at 150 ^oC under argon protection for 6h using 3-aminopropyl triethoxysilane (APTES, 1ml). The surface modified powder was then repeatedly washed by vacuum filtration using ethanol to completely remove toluene, and dried in a vacuum oven at 80 ^oC for 12h.

1.3 Preparation of MWCNT/SiC ceramic composite

The SiC powder and sintering aid were uniformly mixed with MWCNT via heteroaggregation process. Specifically, 1g of modified SiC/sintering aid powder was dispersed in deionized water (100 ml) and the pH of solution was adjusted by hydrochloric acid (HCl) to 2.5 and sonicated for 30 min. Meanwhile, acid treated MWCNTs were dispersed in deionized water (1mg/ml, pH between 2-3) and dripped into the well-dispersed SiC/sintering aids suspension under magnetic stirring. MWCNTs and SiC/sintering aids powder were quickly assembled and settled down to the bottom, leaving clear water as supernatant. The precipitated MWCNT/SiC/sintering aid mixture was collected by vacuum filtration, and then dried at 80 ^{o}C in vacuum oven for 12 h.

1.4 Sintering of MWCNT/SiC ceramic composites

MWCNT/SiC ceramic composite was consolidated by spark plasma sintering (FCT, German). The MWCNTs/SiC/sintering aid powder was loaded in the graphite mold and sintered at 1800 ^{o}C for 5 min with heating rate of 130 ^{o}C min^{-1} in vacuum (6 Pa). The temperature raised up to 1000 ^{o}C under uniaxial pressure of 50 MPa, and then gradually increased to 70 MPa till the temperature reaching 1800 ^{o}C.

1.5 Characterization

X-ray diffraction (XRD) was performed by using, Cu Kα radiations with scanning range of 10-90^{0}). The quality of MWCNTs was characterized by Raman spectroscopy (Tokyo Instruments Co.). The scanning electron microscopy observation was conducted by field emission electron microscopy (FE-SEM, JEOL, JSM-6700F). Transmission electron microscopy (TEM) and high resolution TEM (HRTEM, JEOL 200CX) were used to investigate the microstructure of samples. Advanced ultrasonic material characterization system (UMS-100, France) was used to measure the

Young's modulus and Poisson's ratio. The Vickers hardness was determined by using a load of $5Kg_f$ for 5s. The indentation fracture toughness was calculated by following Anstis equation:

$$K_{IC} = 0.016(E/H_v)^{0.5}PC^{-1.5} \quad (S1)$$

, where E is young's modulus, H_V is Vickers indentation hardness, P is the load, and C is half crack length. Modified small punch (MSP) method was used to calculate the strength of MWCNTs/SiC composite by the following equation:

$$\delta = {}^{3P}/_{2\pi t^2}\ [1 - [(1-\gamma^2)/4)].\left({}^{b^2}/_{a^2}\right) + (1+\gamma)\ln({}^{a}/_{b})] \quad (S2)$$

where P is the load, thickness of sample is t, γ is the Poisson's ratio, a and b are the radius of the hole of the mold and the radius of the tip of cylindrical punch, respectively.

The in-situ pull-out test was performed inside an SEM chamber (JEOL JSM 6600 SEM, Japan) . An AFM cantilever (NANOSENSORS, PPP-ZEILR, nominal force constant 1.6 N/m) was mounted at the end of a piezoelectric bender (ceramic plate bender CMBP01, Noliac) on the X-Y linear motion stage. The tensile fracture surface of composite was uniformly coated by Au spattering and was then fixed on the opposite Z linear motion stage. Electron-beam-induced deposition (EBID) was employed for bonding a selected individual MWCNT onto the cantilever tip with n-docosane (C22H46, Alfa Aesar).

The in-plane dc conductivity was measured by Van der Pauw method using a Hall measurement system (Lakeshore 8400 Series HMS, USA). The out-of-plane ac conductivity was tested on a

broadband dielectric and impedance spectrometers (concept 40, Novocontrol Technologies, Germany) at 1 Vrms/100 mA and at room temperature in frequency range of 50–105 Hz.

2. **Computational methods**

All density functional theory (DFT) calculations are performed in the Vienna Ab initio simulation package (VASP)[1-2]. The generalized gradient approximation (GGA) with the Perdew–Burke–Ernzerhof exchange–correlation function and a 400 eV cutoff for the plane-wave basis set are employed[3]. The projector-augmented plane wave (PAW) is used to describe the electron–ion interactions[4]. All the calculations are spin-polarized and the convergence threshold is set as 10^{-4} eV in energy and 0.05 eV/Å. The k-point sampling of the Brillouin zone is obtained using a 2×4×1 by the Monkhorst–Pack scheme. Denser k-points (4×8×1) are used for the electronic structure calculations. The vacuum slab of 15 Å is inserted in the z-direction for surface isolation to eliminate periodic interaction. For the systems the interaction energy ΔE of the interface between the MWCNTs and SiC/YAP is defined as

$$\Delta E = \frac{E_{total} - E_{MWCNTs} - E_{SiC/YAP}}{N} \qquad (S3)$$

where the subscripts total, MWCNTs, SiC and YAP denote the total energies and the energies of the corresponding substances, while N is the number of carbon atoms of MWCNT at the interfaces.

References

1 Kresse G., Furthmüller, J. *Comp Mater Sci* 1996, **6**, 15.

2 Kresse G., Furthmüller, J. *Phys Rev B* 1996, **54**, 11169.

3 Perdew J., *Phys Rev Lett* 1996, **77**, 3865.

4 Blochl P., *Phys Rev B* 1994, **50**, 17953.

3. **Supplementary figures**

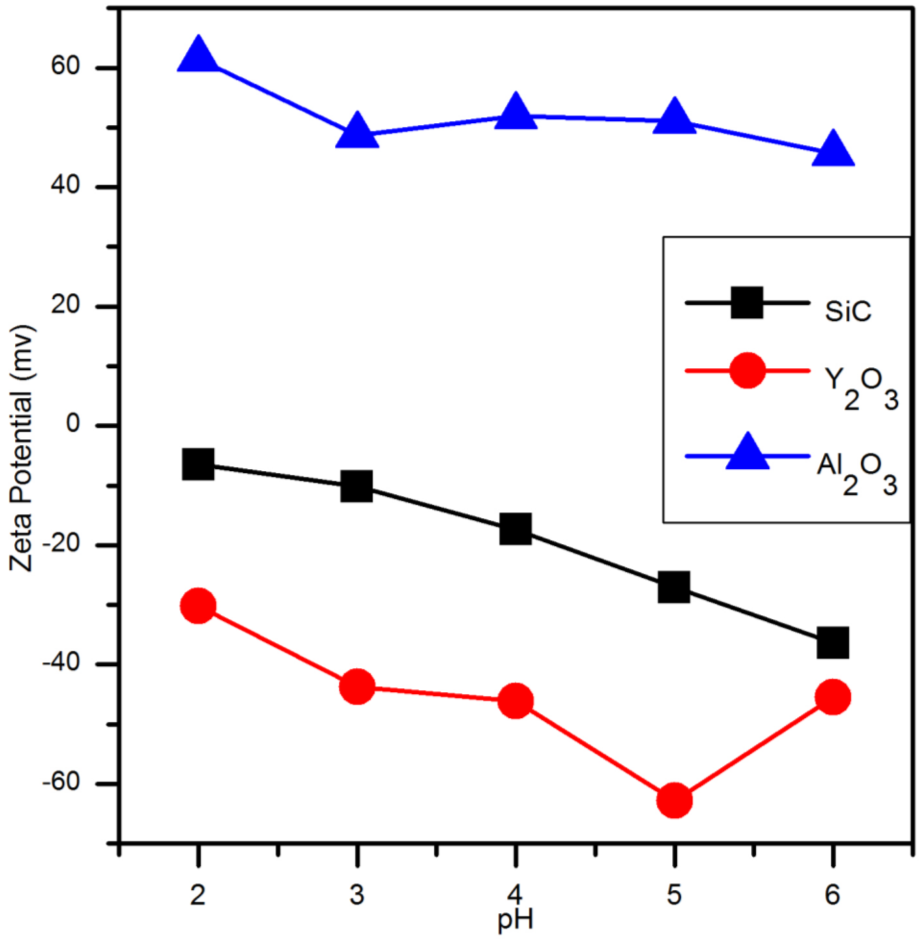

Figure S1 Zeta potential of SiC and Y_2O_3 was negative values and Al_2O_3 has positive zeta potential before modification.

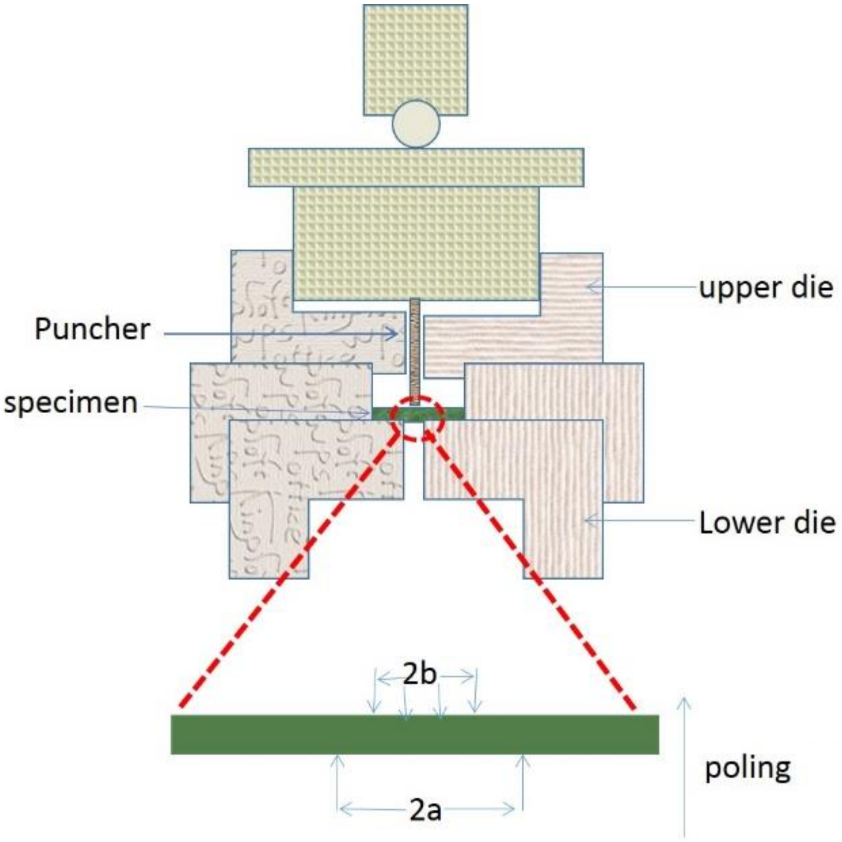

Figure S2 Schematic drawing of MSP-testing apparatus and model for strength calculation.

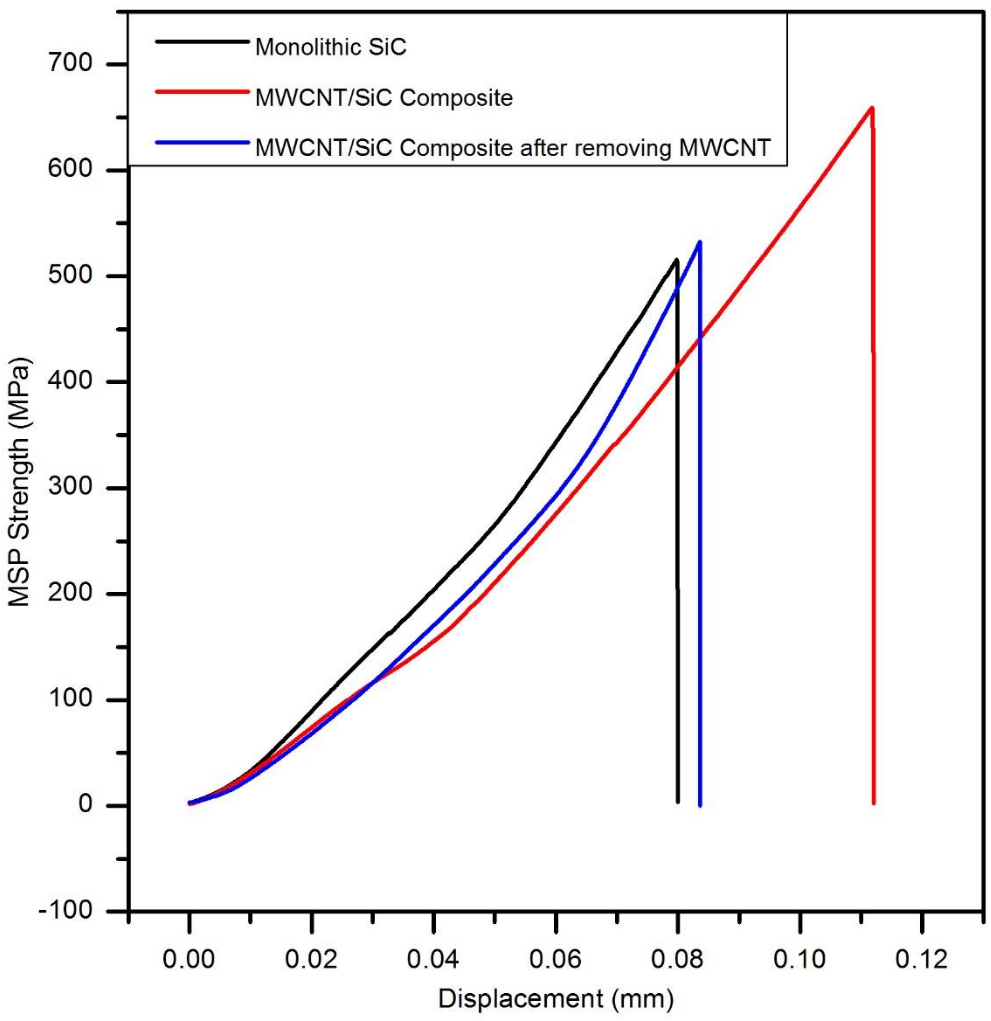

Figure S3 The MSP strength-displacement relation for monolithic SiC, 3 wt% MWCNT/SiC composite and the composite with removed MWCNT. For removing MWCNT, the composite was annealed in air at 800℃ for 2h.

Table S1 A full list of the MWCNTs tested in the pullout experiments

Sample	Outer diameter (nm)	Inner diameter (nm)	Breaking force (μN)	Nominal strength (GPa)	Pullout Length (nm)	Interfacial Strengh (MPa)	Failure modle
1	64.3	-	6.8196	2.1	374	90.3	Pull-out
2	39.618	-	5.5958	4.53928	1734	25.9	Pull-out
3	40.183	-	4.0398	4.4125	961	46.1	Pull-out
4	75.25	-	5.64	1.268	-	-	Fracture
5	27.403	-	2.2815	3.868	-	-	Fracture
6	59.327	-	1.6074	0.5815	-	-	Fracture
7	25.11	-	1.6113	3.25	-	-	Fracture
8	54.802	8.12	8.063	3.4183	-	-	SIS
9	62.622	16.304	9.4532	3.098	-	-	SIS

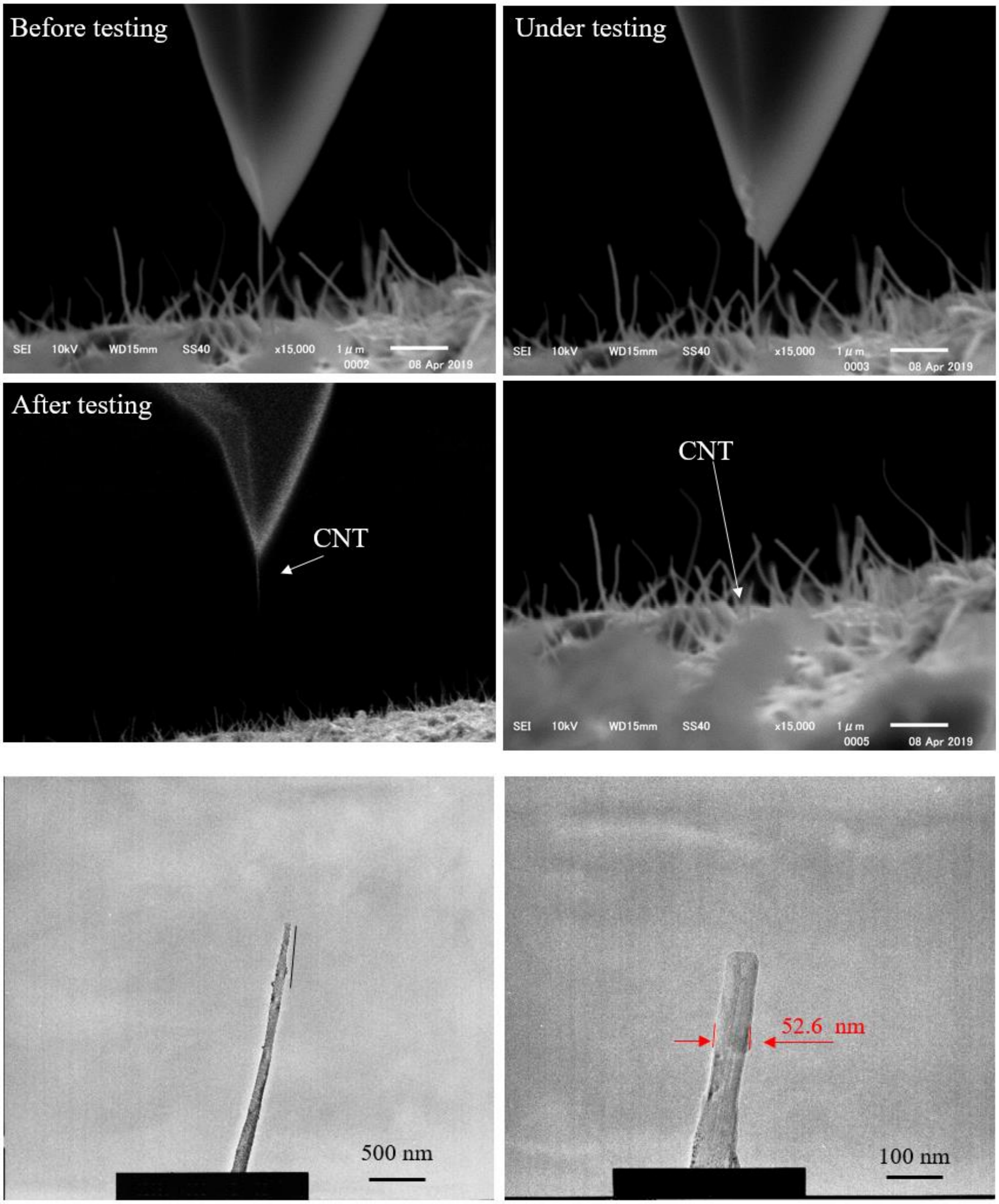

Figure S4-1 SEM and TEM images of sample 1 in Table S1 for in-situ pullout test.

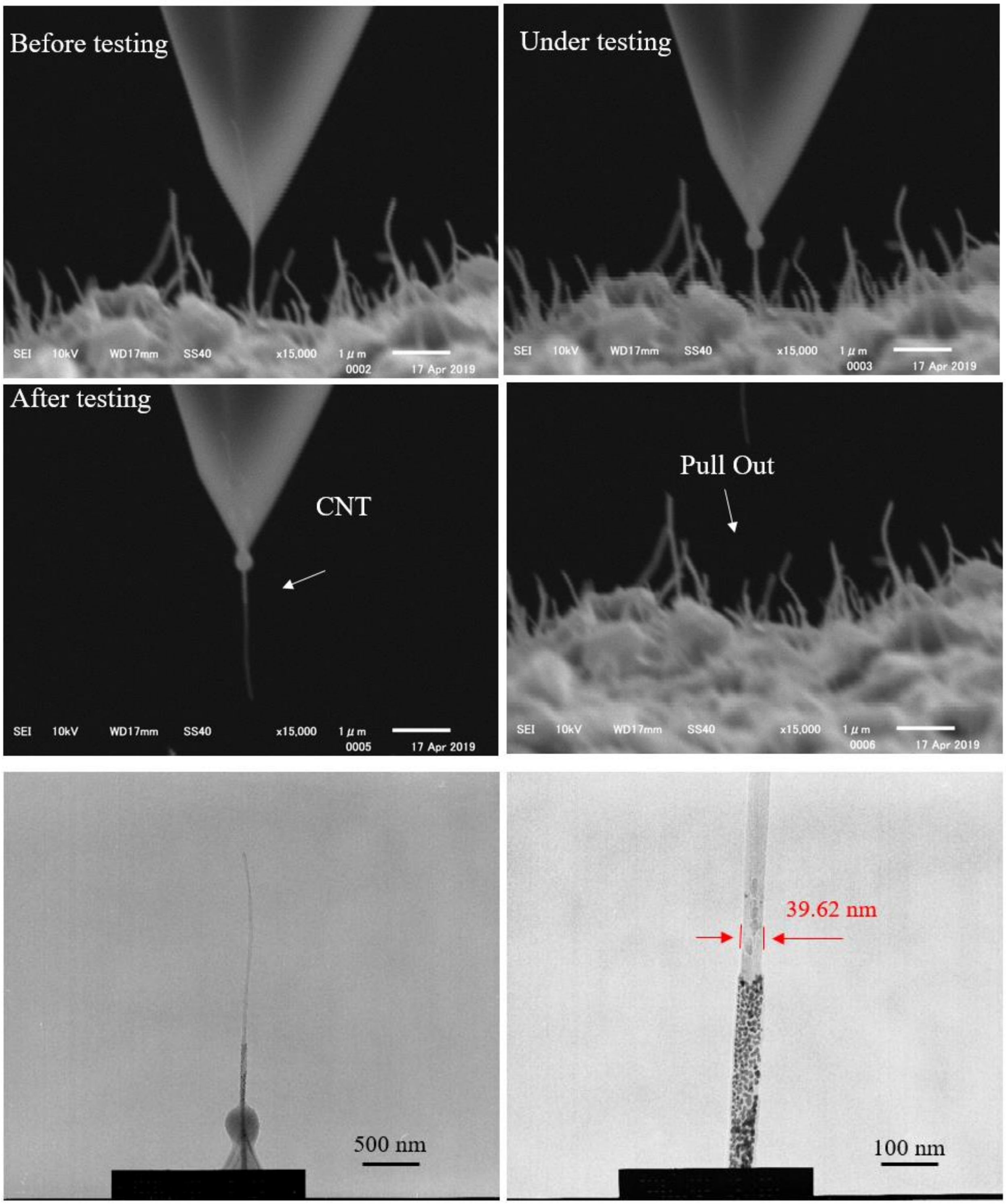

Figure S4-2 SEM and TEM images of sample 2 in Table S1 for in-situ pullout test.

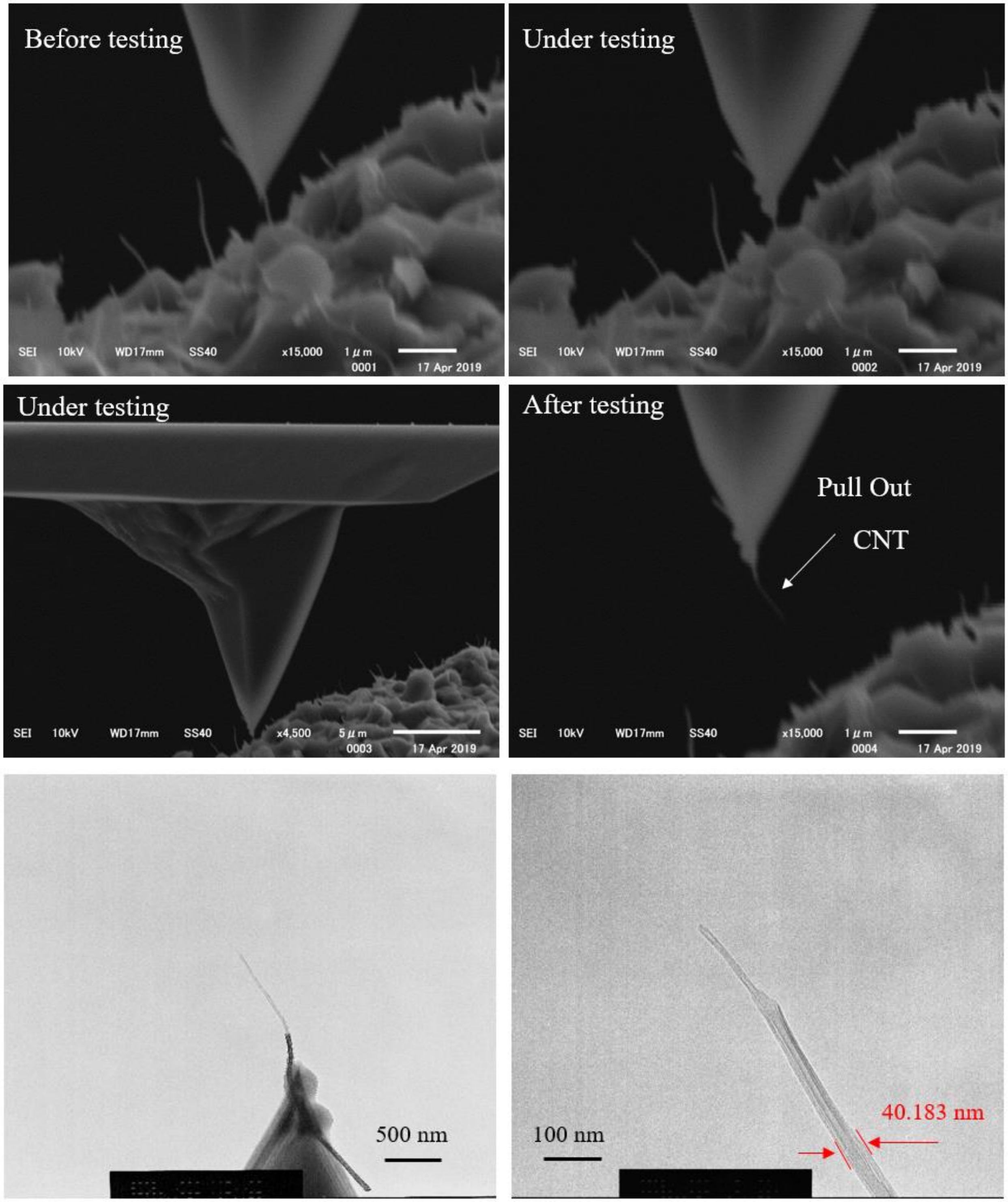

Figure S4-3 SEM and TEM images of sample 3 in Table S1 for in-situ pullout test.

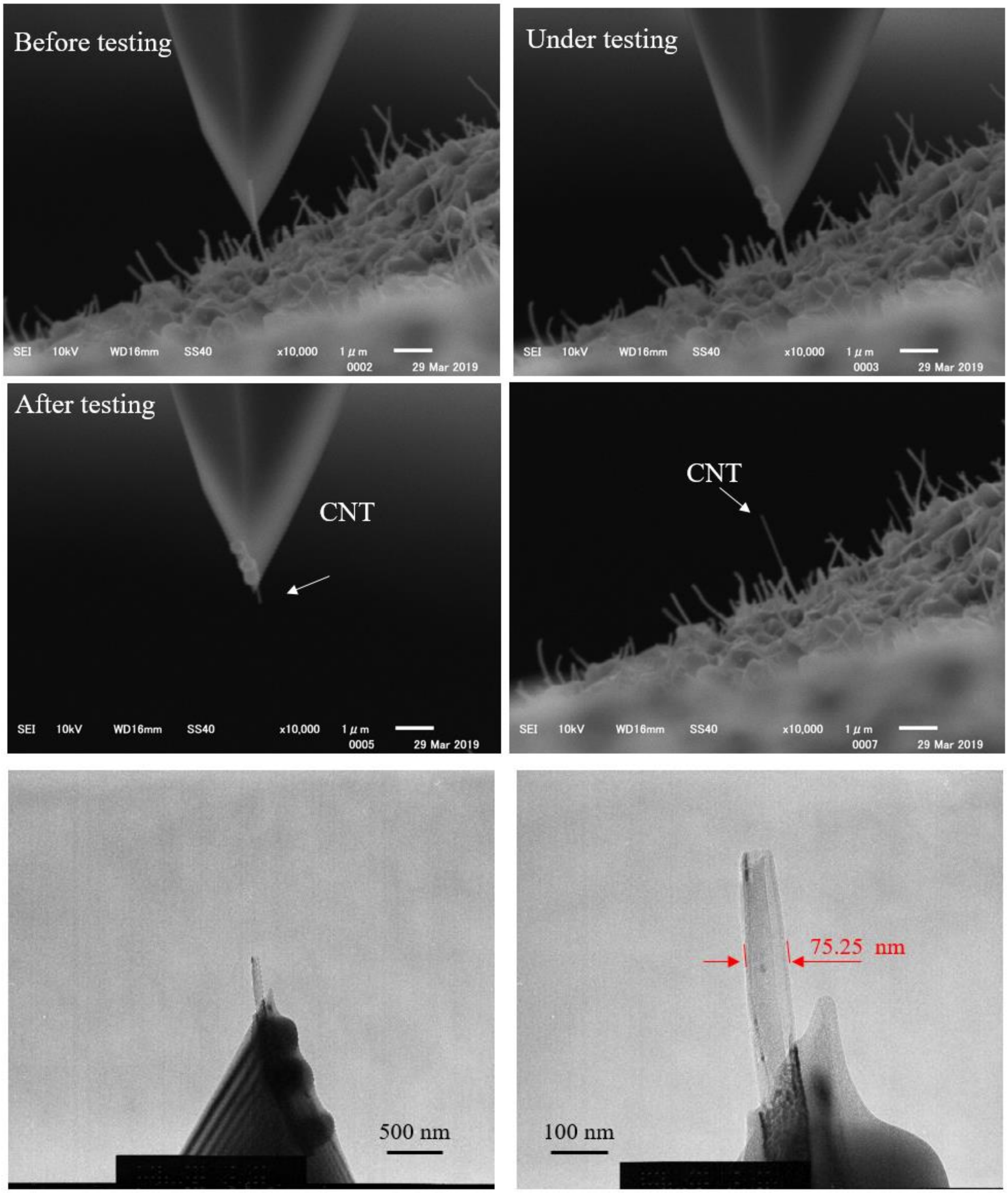

Figure S4-4 SEM and TEM images of sample 4 in Table S1 for in-situ pullout test.

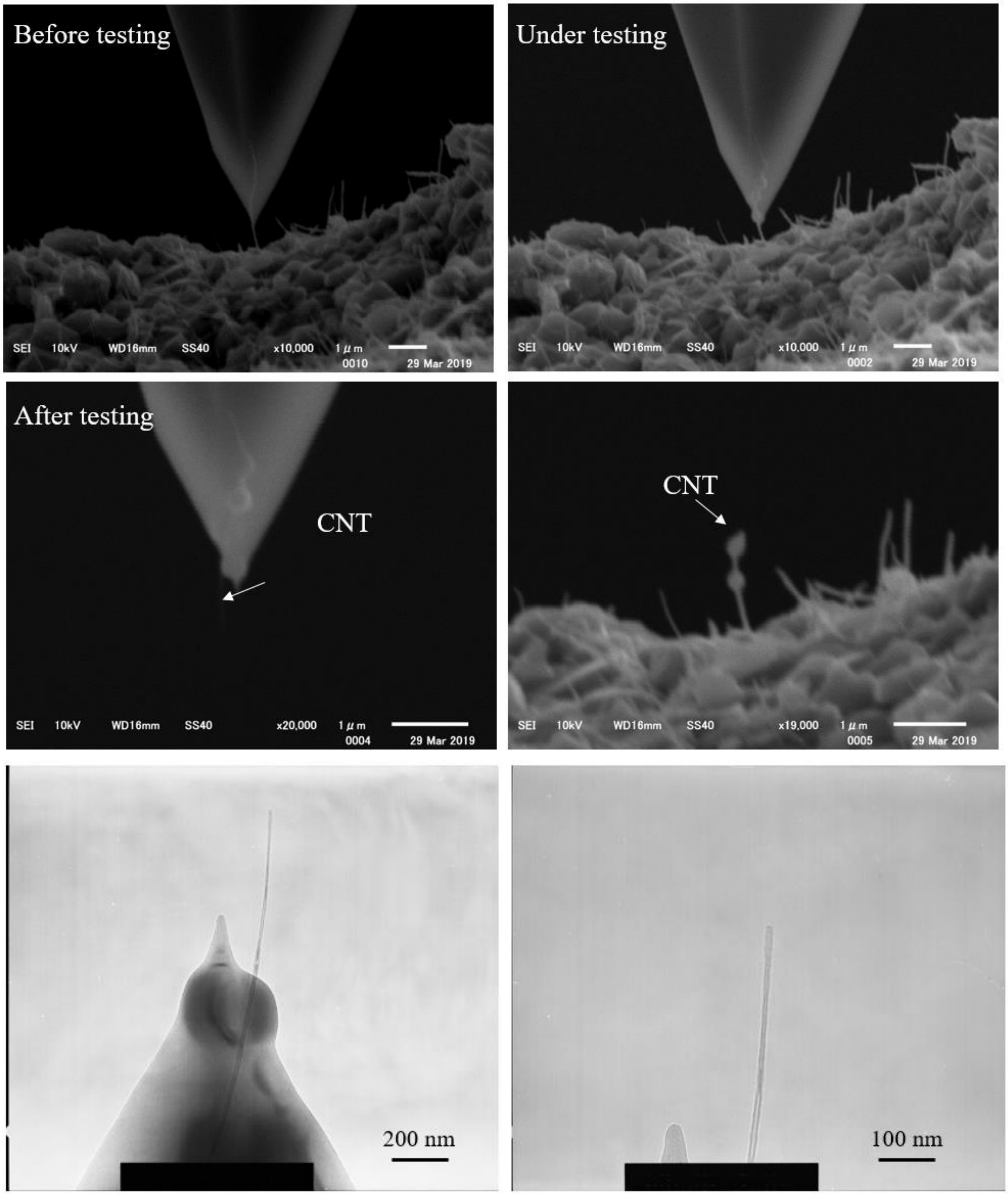

Figure S4-5 SEM and TEM images of sample 5 in Table S1 for in-situ pullout test.

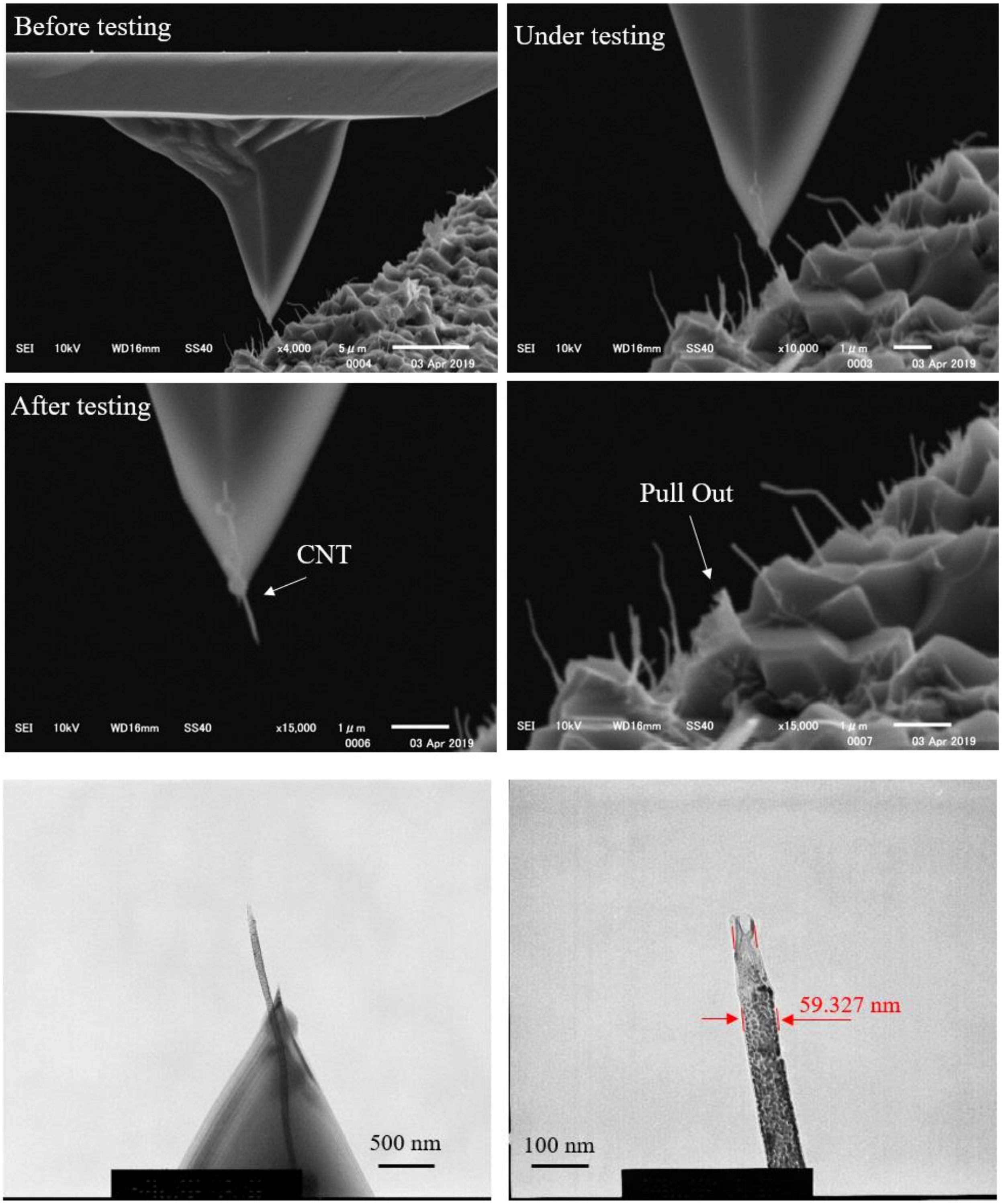

Figure S4-6 SEM and TEM images of sample 6 in Table S1 for in-situ pullout test.

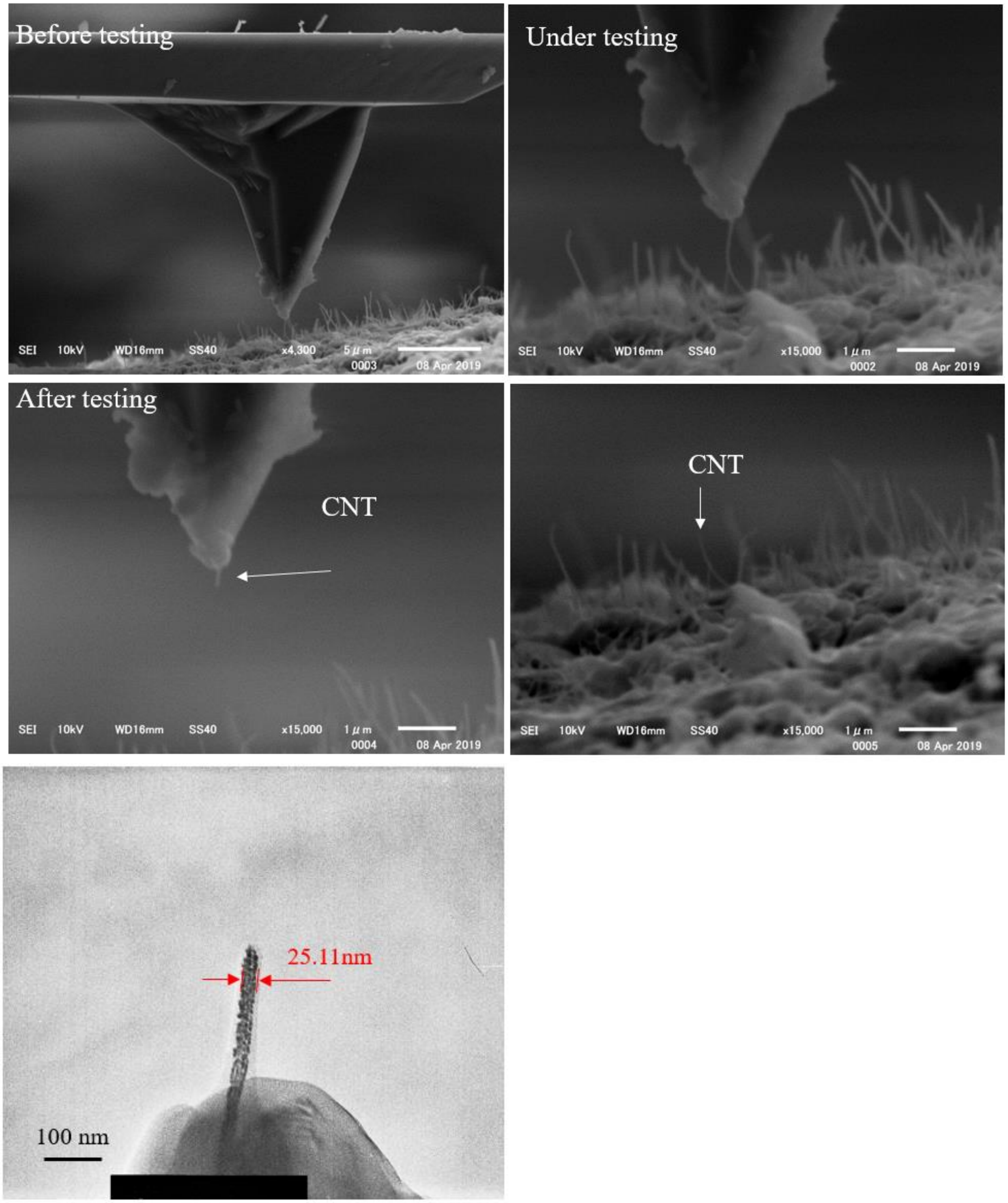

Figure S4-7 SEM and TEM images of sample 7 in Table S1 for in-situ pullout test.

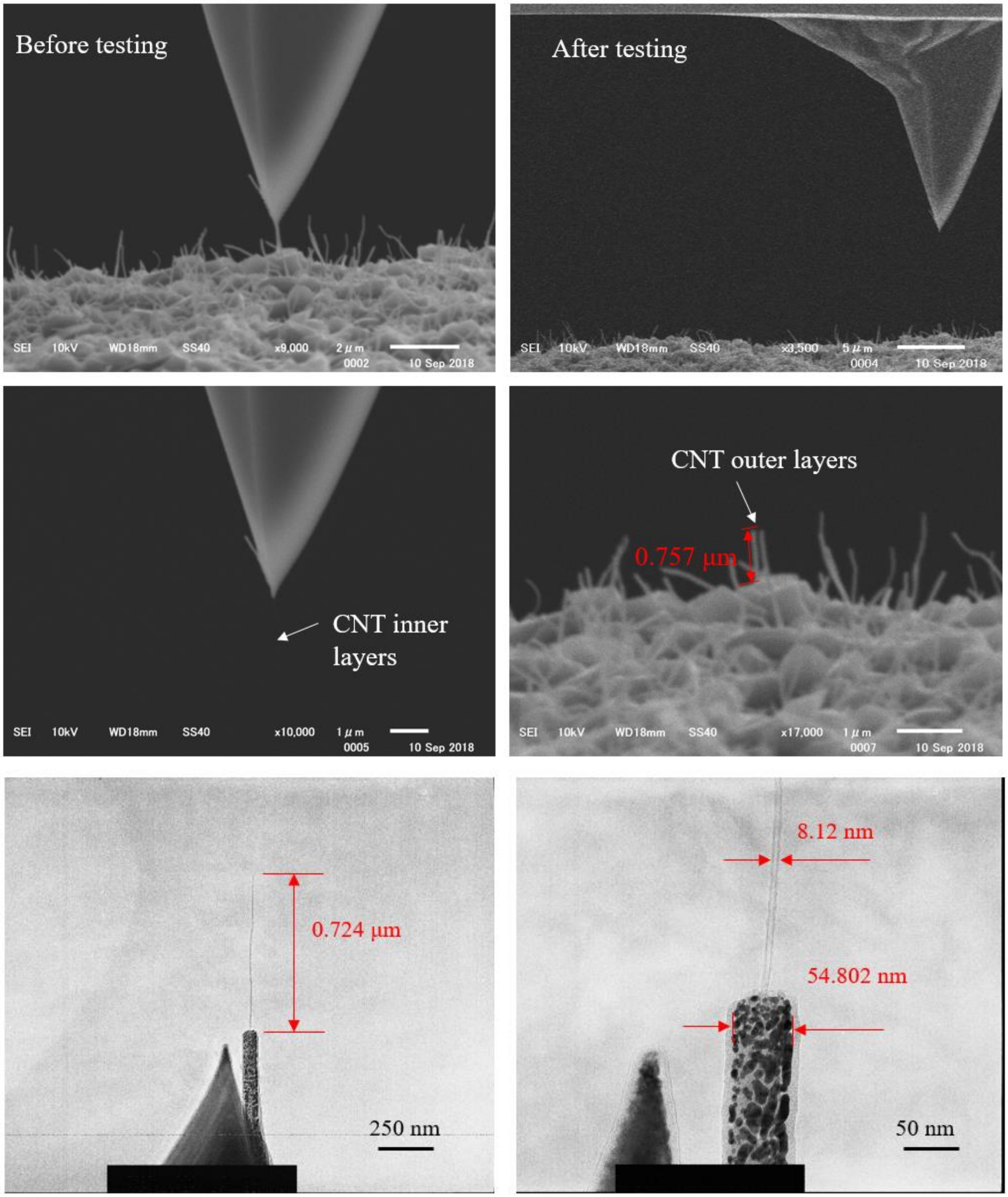

Figure S4-8 SEM and TEM images of sample 8 in Table S1 for in-situ pullout test.

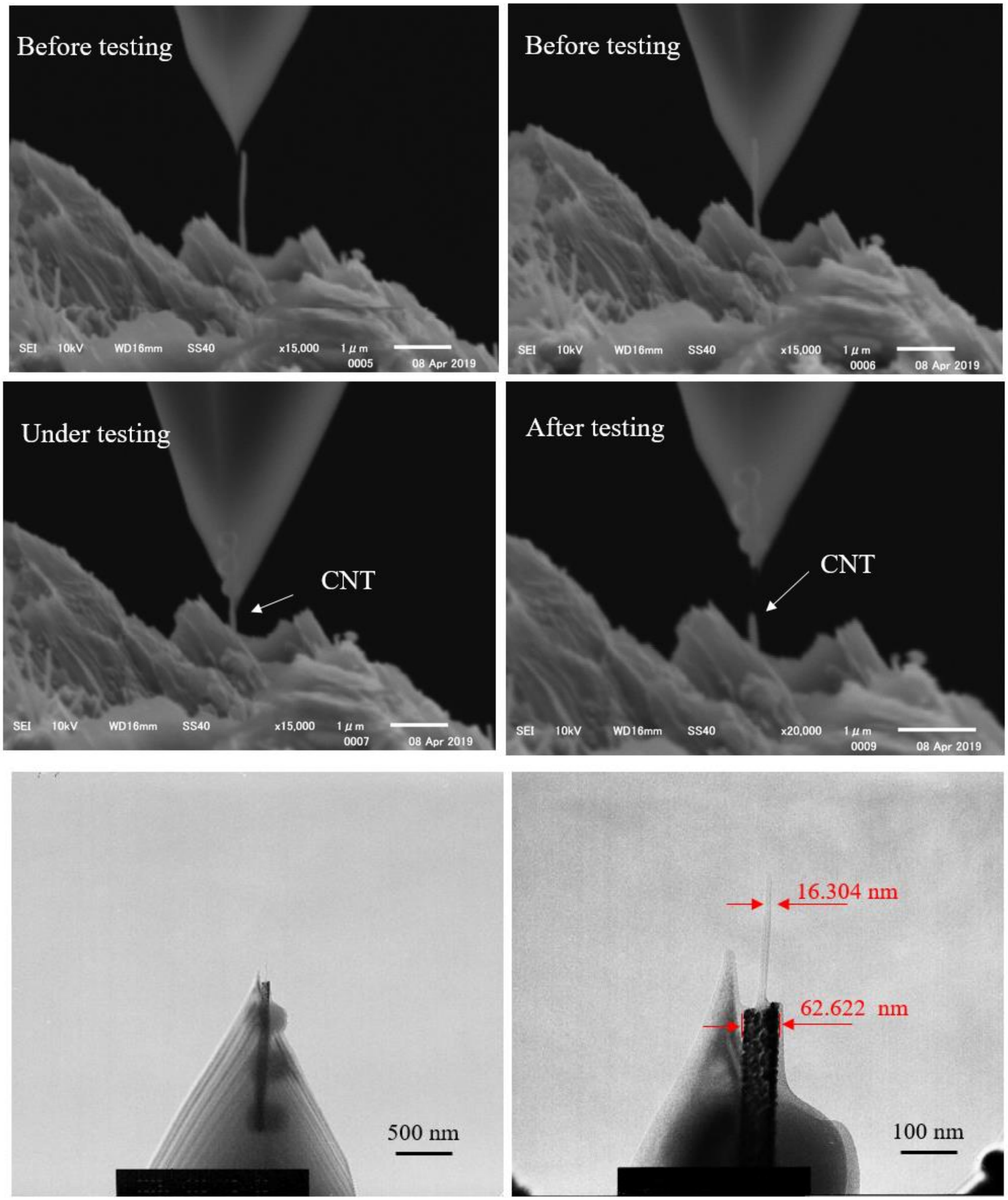

Figure S4-9 SEM and TEM images of sample 9 in Table S1 for in-situ pullout test.

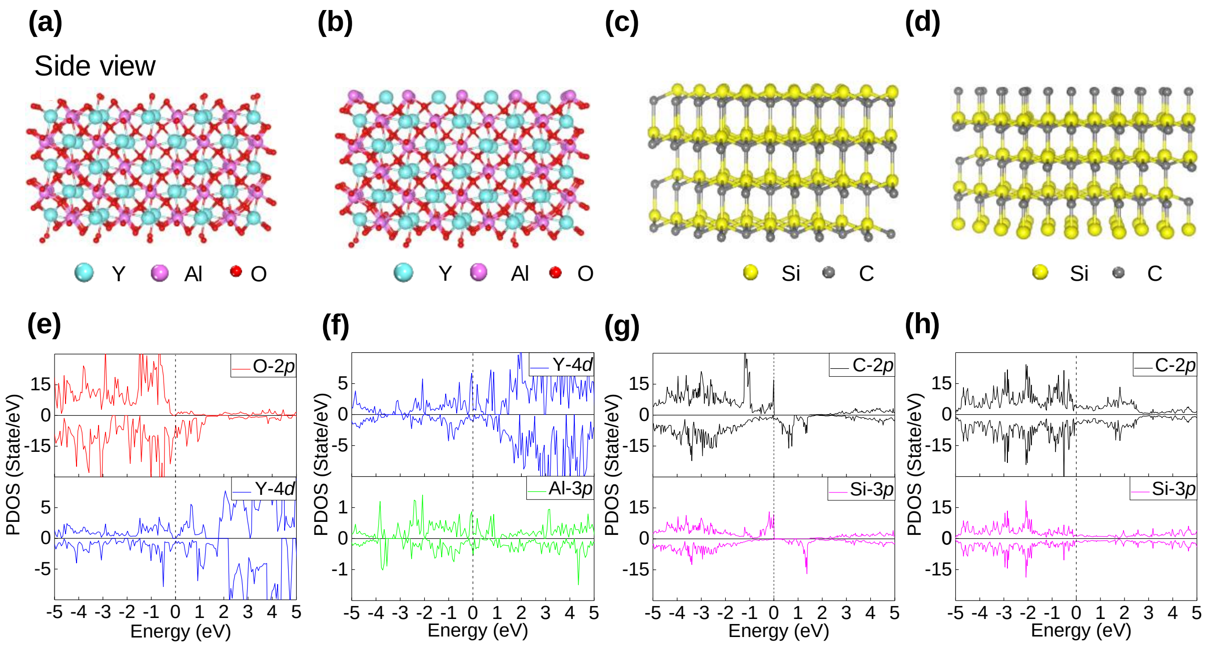

Figure S5 Optimized structure and electronic structures of multiple YAP and SiC surface. (a-d) The optimized structure of surfaces: YAP (002)-O (O terminated), YAP (002)-Y (Y terminated), SiC (0001)-Si (Si terminated), SiC (0001)-C (C terminated). (e-h) The corresponding PDOS of O-2*p* and Y-4*d* orbital of YAP(002)-O, Al-3*p* and Y-4*d* orbital of YAP(002)-Y, C-2*p* and Si-3*p* orbital of SiC(0001)-Si and SiC(0001)-C respectively.

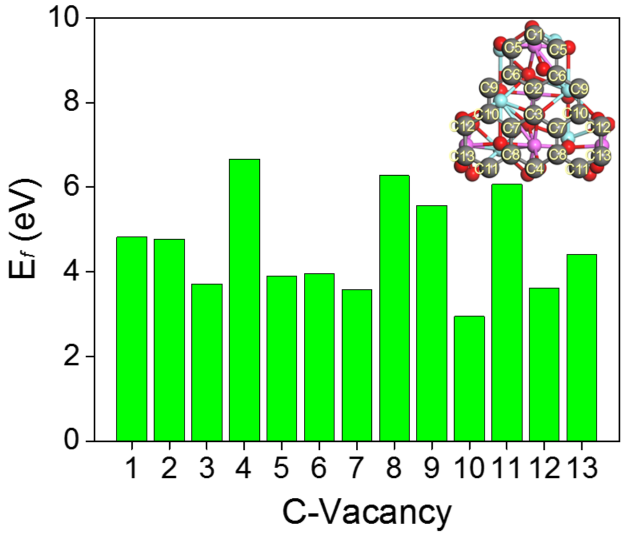

Figure S6 The formation energies (E_f) of proposed carbon vacancy (Vc) in MWCNT/YAP (002)-O Interface. The formation energies are calculated as $E_f = E$ (*MWCNT_n*Vc) + *nE*(C)-*E* (*MWCNT*). The average formation energy of carbon vacancy is defined as E_f/n, where *E* (*MWCNT*) and *E* (*MWCNT_x*Vc) are the total energy of without or with C vacancy of the interface model respectively, *E*(C) is the energy of one carbon atom of MWCNTs in the bulk, n is the number of carbon vacancy in the interface model.

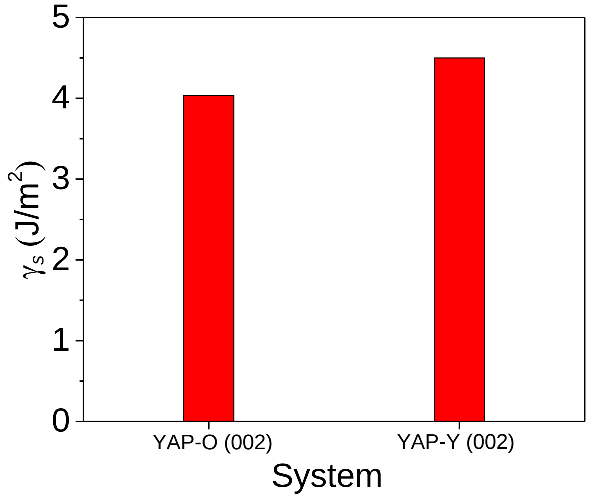

Figure S7 Calculated surface energies (γ_s) of YAP-O (O terminated (002)) and YAP-Y (Y terminated (002)) structure. The surface energies are calculated as $\gamma_s = \frac{1}{2A}(E^{unrelax}\text{-}nE_b) + \frac{1}{A}(E^{relax}\text{-}E^{unrelax})$. Where E^{relax} and $E^{unrelax}$ are the total energy of (unrelaxed) the slab model for the surface, respectively. E_b is the energy of one atom of the material in the bulk, n is the number of atoms in the slab model. A is the total area of the surfaces in the slab model.

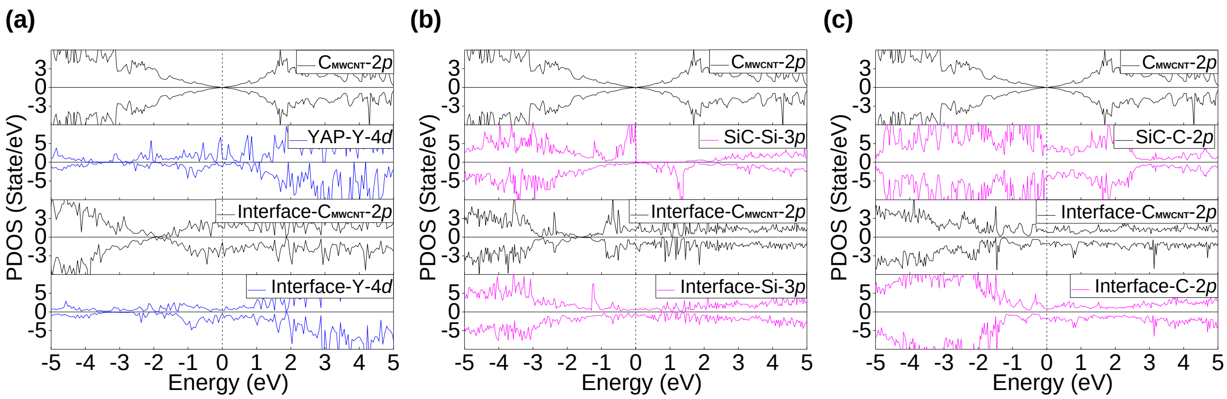

Figure S8 The electronic structures of multiple MWCNT/YAP (002)-Y, MWCNT/SiC(0001)-Si, MWCNT/SiC(0001)-C interface. (a-c) The corresponding PDOS of C_{MWCNT}-2*p* and Y-4*d* orbital of YAP(002)-Y (a), C_{MWCNT}-2*p*,Si-3*p* and C-2*p* orbital of SiC(0001)-Si and SiC(0001)-C(b, c), respectively.
